# Medical and Physical Intelligence

**Published:** 1824-09

**Authors:** 


### MEDICAL AND PHYSICAL INTELLIGENCE.

### ANATOMY.

1. *Structure of the Gall-Bladder.*—M. Amusat lately exhibited to the Academy of Medicine several anatomical preparations of the biliary canals, demonstrating the true mechanism of the reflux of the bile from the ductus choledochus into the gall-bladder. M. Amusat has discovered, and shown, the existence of a spiral valve, a sort of Archimedes' vice reversed, which the neck of the gall-bladder is provided with.—(*Revue Medicale*, Juin.)

### MORBID ANATOMY.

2. *State of the Blood in Jaundice.*—M. Chevreul observes, that there are some peculiarities in the blood of new-born children who die of the disease called skin-bound (*induration*). If the skin of these subjects is incised, a yellow liquid escapes, composed of albumen, a colouring matter of an orange-red, and one of a green colour; and these matters are also found in the bile of these infants. The blood of children with jaundice differs also much from that of healthy children, as far as regards the serum; its composition and colour being the same as above mentioned.—(*Journal de Pharmacie*, Juin.)

3. *Ossifications of the Arachnoid Membrane.*—MM. Cullerier and Maingault relate the case of a maniac, in whose brain several scrofulous cysts and abscesses were found, as well as many ossifications of the arachnoid membrane. The patient had complained during life of a very disagreeable smell.—(*Revue Medicale*, Juin.)

4. *Rupture of the Bladder.*—Dr. Fix, of Berne, reports the case of a young lady, aged twenty, of a lymphatic temperament, who had suffered, from the age of puberty, with various symptoms of debility: her face was remarkably pale; and, though she menstruated regularly, the discharge was scarcely coloured. After having languished in this way for some time, symptoms of intestinal inflammation supervened. Constipation and retention of urine were troublesome; the abdomen was tense, tender to the touch; the pulse small and contracted, and sixty in the minute. Glysters lessened the volume of the abdomen, but did not decrease the pain. The urine was passed frequently, in small quantities, and loaded with mucus; thirst great, and appetite quite gone. Death soon followed. On opening the body, a serous effusion, to the amount of ten or twelve pounds, was found; traces of inflammation also were perceived on the small intestines; but the bladder was the principal seat of disease: it was reduced to a thin membrane, resembling a mucous net-work, and was torn throughout nearly the whole of its extent.—(*Journal der Pract. Heilkunde.*)

### PHYSIOLOGY.

5. *Effect of Castration in certain Animals.*—M. Faneau Delacour, of Souzay, has performed a number of experiments upon sheep and pullets, with a view of determining the effect of castration upon the animal economy, conceiving that the loss of organs so important as the testicles, could not take place without materially affecting the health; which opinion was strengthened by considering the sudden evils often arising from more trifling causes,—such as the disappearance of eruptions, or the drying up of a long-established ulcer.

M. Delacour had eighty pullets castrated in his presence: eleven of these immediately exhibited well-marked signs of cerebral affection, and in three others the symptoms were observable, but not to so great a degree. Of eight which became mad, four of the worst, as well as two out of three which were threatened with apoplexy, were cupped upon the rump, and an actual cautery applied on each side of the cupping-glass; and in the four first instances, a cautery was also applied on the head. All these recovered: whereas, one left entirely to the efforts of nature died on the third day, the brain exhibiting the strongest marks of inflammation.

The same phenomena were observable among a flock of sheep, and in a greater proportion. The same remedies were made use of in seven of these animals, and they all recovered on the day the cauteries were made: whereas, two left entirely to nature died,—one on the fourth day, with all the marks of madness; the other on the second day, in a state of coma. The examination of the heads showed, in the first instance, a violent state of inflammation of the brain and its membranes: the brain of the second was softened, and the ventricles filled with a fluid resembling the white of an egg a little coloured.—(*Journal Universel des Sciences Medicales,* Juin.)

6. *Wound through the Chest, piercing the Diaphragm and Stomach.* —On the 7th of May, 1824, a man, about twenty years of age, on the evening of his wedding-day, plunged a very sharp cook's knife in the interval between the sixth and seventh rib, on the left side, and towards the sternum. He immediately fainted away. The medical men who were called in, judging from the situation and direction of the wounds, and the marks of blood upon the knife, that it had penetrated about two and a half inches; the symptoms announced an internal hemorrhage, which appeared to cease for a time: it, however, soon recurred, and the man died thirty-four hours after committing this desperate act. The body being opened, the following appearances presented themselves:—The wound had penetrated the cavity of the thorax for the space of an inch, but the lung was not wounded; yet there was a considerable effusion of blood on that side, arising from the wound of an intercostal artery. At the spot where the diaphragm was wounded, a sound was stopped in consequence of the protrusion of a portion of omentum. In the abdomen, the stomach was found pierced to the extent of three lines, at its anterior and superior part; and an effusion of blood had taken place into the colon, extending to the hypogastric region.

The conclusion which the reporter of this case (Dr. Millet) draws from this examination, is that it tends to confirm the experiment of Dr. Williams, and goes to prove that the lungs do not, in their natural state, fill the interior of the pleura in the act of respiration; for, had that been the case in the instance above related, the lung could not have escaped without being wounded.—(*Journal Universel,* Juin.)

---

### PATHOLOGY.

7. *Elongation of the inferior Extremities.*—MM. Richerand and Cloquet relate the case of a patient of the hospital of St. Louis, whose lower limbs admit of being alternately lengthened and shortened to the extent of three or four inches. These gentlemen explain the circumstance by supposing that the heads of the ossa femora are destroyed, as well as the sides of the cotyloid cavity. The patient is fifty years of age; he walks with difficulty, but without pain. The limb upon which he stands becomes shortened, and the great trochanter touches the crusta ilii; the limb which he raises, on the contrary, becomes lengthened to its natural state, and alternately shortened upon making another step. The patient is afflicted with several exostoses of the bones of the pelvis, as well as many ossific tumors in the substance of the muscles.—*(Revue Medicale,* Juin.)

8. *Cure of external Hydrocephalus by Puncture.*—Dr. Fenoglio relates the case of a child, eighteen months old, who fell from a balcony fifteen feet from the ground: the left parietal bone was depressed, but there was no fracture, and not a drop of blood escaped from the nostrils; the left humerus, and the bones of the fore-arm, were fractured. The parietal bone resumed its usual form in a few hours. On the day following the accident, violent fever came on: the breathing was stertorous, and the skin was burning hot; the lower extremities were cold, and there was a trembling motion of the right hand. Bleeding by leeches was resorted to, and ice applied to the head; and the fever was relieved. At the end of the fourth day, however, a fluctuating tumor was perceived at the posterior fontanelle, and which, being pressed upon, disappeared, but returned when the pressure was removed. In proportion as this tumor increased externally, the child became more lively; but, as Dr. Fenoglio justly saw the danger which threatened, in consultation with Dr. Giordano and Professor Rossi, it was determined to wait some time before any attempt was made to remove the swelling, considering it to be the product of extravasation only. After the seventh day, however, they changed their opinion as to its nature, and a small puncture was made at its most depending part, and a corrupted and fœtid lymph was evacuated. The infant immediately fell asleep, and slept for eight hours; but awoke at the end of that time with renewed fever, and the symptoms previously described. Leeches were applied to the left foot, an opening medicine administered, and a strong infusion of digitalis ordered. *(Neither the strength nor doses of this infusion are mentioned.)* In the evening, the fever was diminished. The opening into the tumor was not closed, and a fluid escaped from it drop by drop, but so slowly, that it was only known by the moisture of the pillow.

The intellectual and physical faculties of the child improved rapidly; the bowels acted freely; and this amendment went on from day to day, so that the parents conceived her free from danger. At the end of the second week, however, on a sudden, the tumor ceased to discharge; there was suppression both of fæcal evacuations as well as of the urine, and the former symptoms again recurred. Leeches were again applied to the ankles, castor-oil given so as to purge, and the digitalis again had recourse to, with so good effect, that in about eleven days the hydrocephalus had entirely disappeared.

Another severe attack was experienced after this, preceded by vomiting, and accompanied with convulsions of the whole body, but which were relieved by the same means; and the patient finally got well.—*(Journal Universel,* Mai.)

### THERAPEUTICS.

9. *Belladonna a Preventive of Scarlet Fever.*—It has been long known that Dr. HAHNEMANN, of Leipsic, has asserted the above fact; but, since the year 1818, several practitioners in the north of Europe have repeated these experiments, and they find them founded in truth. The first of these, Dr. BRENDT, of Custrin, affirms that all who employed this remedy escaped the infection; and his account is corroborated by Dr. MUHSBECK, of Demmin, in Western Pomerania, who says he has used it for seven years, and with equal success; and he administered it to all those who dwelt in the houses where scarlet fever prevailed, continuing its use until desquamation of the cuticle had taken place in those attacked. Dr. DUSTERBOURG, of Warbourg, has also published an account of a series of experiments, confirming these statements; and several subsequent Memoirs have appeared, all equally corroborative of this virtue in the belladonna. The formula generally recommended is a solution of two grains of the extract in an ounce of some distilled water; and to children from one to ten years of age, from one to five drops of this solution is given four times in the day; from ten years of age and upwards, from six to ten drops is given, also four times in the twenty-four hours.—(*Revue Medicale*, Juin.)

10. *Nitrate of Silver in Chorea.*—M. PRIOU, of Nantes, has published two cases of chorea, cured by the nitrate of silver; which he, however, considers as a remedy quite new, and first employed by himself in these cases. The first case is that of a female, seven years and a half old, who had tried all the usual remedies in vain. The following formula was prescribed:—℞. Argent. Nitrat. gr. vj. Ext. Opii ʒj. Moschi ℈ij. Camphoræ ℈iv. divided into ninety-six pills; and one was at first taken morning and night. In fifteen days, all the symptoms had disappeared. The disease afterwards returned; but the same remedies cured it again. Sixty-nine pills were taken during the treatment. The patient is now fifteen years and a half old, and remains well.

The second case is also a female, seven years of age, who was seized with chorea suddenly after a fright. After employing various medicines, the cold bath, &c. without success, or with only partial relief, the above-named pills were given, one morning and evening. After the fourth day, in consequence of the perceptible amendment, the parents of the child gave eight pills in the day, without informing M. Priou. They were afterwards continued for a month, at the rate of two, and then one, in the day; when the patient was cured, and has had no relapse.—(*Journal General de Medecine*, Juin.)

11. *Poisoning by Hydrocyanic Acid.*—Dr. HELLER, in a small pamphlet, lately published, on the above subject, objects to the use of certain excitants, such as the oil of turpentine and strong coffee; as, he says, they are always useless when the dose of this acid has been large enough to stop the animal functions; and that they are positively hurtful when the quantity has been so small as only to produce those symptoms which terminate of themselves; and finally, that, in these cases of poisoning, the only stimulants necessary to be used are ammoniacal or etherial frictions, the open air, acidulated drink, motion, and exercise.—(*Journal de Pharmacie*, Juin.)

---

### SURGERY.

12. *Operation of Lithotomy*.—M. DUPUYTREN has lately performed this operation in a new manner, and with a new instrument. The operation may be called the transverse operation, and the instrument a double *lithotome caché;* the instrument having, in fact, two blades, so disposed as to cut both left and right at the same time, on withdrawing it from the bladder. The sound is introduced, and the membranous portion of the urethra divided in the usual manner. The lithotome is then introduced into the bladder; it is opened, and, on withdrawing it, the prostate gland is divided so as to be cut in two halves, the one anterior, the other posterior. By this method, the vasa deferentia, the rectum, and the transverse artery of the perineum, as well as the pudica, are said to be avoided. M. Dupuytren has lately operated on a child, one year old, in this manner, and no accident has followed the operation.—(*Bulletin des Sciences Medicales*, Juin.)

---

### CHEMISTRY.

13. *Detection of Hydrocyanic Acid in the Human Body*.—M. LASSAIGNE has published a long, but very interesting, paper on this subject, of which we can now do no more than give the conclusions deduced from his experiments and observations; which are these—

1st. That, by chemical means, it is possible to recognise, in a distilled aqueous liquor, the presence of hydrocyanic acid, in the proportion of one 20,000th part of the weight of the water.

2d. That, in animals poisoned by this acid, it is possible, at the end of eighteen and forty-eight hours, or even a longer period, to detect its presence.

3d. That it is in the viscera where this substance has been primarily taken, that it is to be discovered.

4th. That, in the brain, heart, and spinal marrow, it has not been possible to detect the most minute quantity, although the odour would induce the suspicion of its presence.

M. Lassaigne is aware that M. ITARD's experiments led him to the conclusion, that hydrocyanic acid might be spontaneously developed in the body; but, as the sense of smell alone has been appealed to as a proof of this fact, M. Lassaigne questions its being correct; especially as M. ROBIQUET has shown that the oil of the lauro-cerasus, which has a strong odour of bitter almonds, does not owe this to the small quantity of hydrocyanic acid it contains, but to a volatile oil. Added to which, M. Lassaigne has carefully examined the evacuations of the human subject; the matters contained in the intestines of various animals, especially of dogs, who have died of inflammatory diseases; as well as animal substances in different degrees of putrefaction, and has never been able to detect the presence of this acid.—(*Revue Medicale*, Juin.)

14. *Analysis of the Male Fern Root.*—M. MORIN, of Rouen, informs us that this root, which is successfully employed as an anthelminthic, owes its virtue to a fatty substance, capable of being converted into a soap, of a nauseous smell resembling that of the root, of a very disagreeable taste, heavier than water. The root contains also gallic acid and acetic acid, some sugar, tannin, starch, and a gelatinous matter insoluble in alcohol or water, some woody matter, and the salts usually found in ashes. M. Morin believes this fatty substance to be formed of a fixed and a volatile oil; but he has not obtained yet sufficient proofs of this.—(*Annales de Chimie*, Juin.)

15. *Analysis of the Upas.*—MM. PELLETIER and CAVENTOU, in a Memoir read at the Royal Academy of Medicine, announce the presence of strychnine in the upas. In the upas anthiar, they have not discovered this substance, but a peculiar deleterious principle, soluble in alcohol and water, and which does not possess alkaline properties. It appears from the result of these gentlemen's observations, that the red colour produced by the action of nitric acid upon strychnine, does not depend upon a vegetable alkali; that the intensity of this colour is in an inverse ratio to the purity of the strychnine, being the effect of a foreign substance, with which the alkali is intimately united, both in the nux vomica and the St. Ignatius' bean.—(*Journal de Pharm.* Juin.)

---

### MISCELLANEOUS.

16. *Question of Infanticide.*—We are requested to state, on the part of Dr. GORDON SMITH, that he is under the necessity of postponing his intention of lecturing, this season, on Political Medicine. His friends are aware how frequently he has, of late, been laid aside; and his health is in too precarious a state to warrant the contraction of so important an engagement with the public. He trusts that the delay which may intervene before he can execute a purpose he has so much at heart, will prove advantageous to the ultimate accomplishment of his wishes.

In the mean time, he is about to occupy himself, more fully than has yet been done by any individual, with the important, but ill-understood, subject of *proving or disproving the vitality of new-born children*,—or, to speak technically, of INFANTICIDE. There is no point in Forensic Medicine on which so much perplexity exists, and for the elucidation of which so little has in reality been done. He is persuaded that this is, in great measure, owing to want of due pains being fairly bestowed; for, whether the existing state of knowledge be estimated positively or negatively, no one, who has at all looked at the question, can be unaware that there is much work to be gone through ere satisfactory conclusions can be formed.

It is, perhaps, impossible for any individual—at least, Dr. S. feels that it is beyond the compass of his own power,—to do justice to the problem; as few who possess the means can be supposed to have the leisure requisite to collect, arrange, and apply the necessary data. But he relies on the zeal and liberality of the profession to aid him in so important an undertaking. He, therefore, solicits from those who may have opportunities of handling the bodies of new-born infants, a few items of information, which he hopes will not cause much trouble, and the aggregate of which he trusts he may be enabled to apply with precision and advantage, so that the truth may be established either the one way or the other; and to its due amount, with regard to certain doctrines that have been bandied about for many years, without any fair or satisfactory estimate as to their practical import.

Simple answers to the following queries form the object of the present application, premising that the subjects chosen must be perfect,—that is, of ordinary development; free from redundant parts, mutilation, disease, or putrefaction; and such as are unquestionably of the class to which they may be assigned. If in any particular instance there should be points in morbid anatomy, which, in certain cases, might greatly assist in coming to appropriate conclusions, they should be stated.

The subjects being classed—first, as STILL-BORN, or *such as have never respired;* and secondly, as VIVI-BORN, or those that have come into the world alive, but have died within twelve hours, the queries may be attended to in the following order:—

Class I.—*Subjects* STILL-BORN.

Required—

1. The sex.
2. Period of gestation when born.
3. How long *known* or *supposed* (as the case may be) to have been dead *in utero.*
4. The cause of death.
5. Nature of the labour.
6. Exterior aspect of the body.
   - *a.* As to colour.
   - *b.* —— integrity.
   - *c.* —— development.
   - *d.* —— formation.
   - *e.* —— marks of violence, ecchymoses, or any peculiarity.
7. Length from the vertex to the under-part of the heel.
8. Point at which the middle length of the body falls, to be given as regards its distance from the umbilicus.
9. Weight of the whole body, prior to any interference with its integrity,—to be accurately given in ounces and fractions, stating the species of weight used.
10. Aspect of the lungs *in situ,* on opening the thorax, and form of the diaphragm.
11. Weight of the lungs, separated from all attachments, avoiding the spilling of contents.

12. Weight of any fluid that may escape from the trachea, on holding the lungs over a vessel in the scale, in an inverted position, but *not squeezing* them; and the fluid described.

13. Result of placing the lungs in a washing-basin of water, first entire, then separately,—i. e. the right and left lung each by itself; noting if there be any difference of buoyancy in either, and which: as also, when cut in pieces, noting any morbid appearances in these organs.

14. Weight of the liver, &c. managed in the same way, with the exception of placing it in water.

15. State of the alimentary canal in regard to contents.

16. State of the urinary bladder.

17. State of the gall bladder.

18. State of the ductus arteriosus and venosus.

19. Colour and consistence of the blood; specifying the part or parts of the body in which the observation may have been made.

Class II.—*Subjects* VIVI-BORN.

Required—

1. The sex.
2. Period of gestation when born.
3. First actions—
    *a.* As to crying, or manifestation of the respiratory process.
    *b.* The state of the umbilical cord.
    *c.* Evacuations *per anum ac urethram.*
4. Cause, manner, and time of death.

Then assume the queries as in the other case.

It is neither expected nor desired that any individual shall take the trouble to furnish *a list.* One case, properly investigated and clearly stated, by an intelligent hand, will be worth hundreds of such as seem to have been collected abroad—one hardly knows how. In order, however, to impart necessary satisfaction as to the authenticity of the materials, it will be essential that those who may be pleased to transmit the result of their inquiries, should verify them with their signatures; and in all cases, where practicable, Dr. S. will scrupulously acknowledge his obligations.

Communications may be forwarded for Dr. Gordon Smith, to the care of Messrs. Underwood, 32, Fleet-street, London; and he leaves the economy of transmission entirely to the convenience and discretion of correspondents.

17. *New Work on the Nerves.*—The papers printed in the Transactions of the Royal Society during the last three years, detailing the Discoveries of the Functions of the Nerves, will be immediately republished, with Notes, and a general Introductory View of the Nervous System, by Mr. CHARLES BELL, Professor of Anatomy and Surgery to the Royal College of Surgeons, and Surgeon to the Middlesex Hospital.

18. MASCAGNI's *Anatomical Plates.*—Dr. GROTANELLI, Professor of Clinical Medicine at Sienna, in a discourse lately delivered before the Royal Institution of Sciences at Paris, observes, that the drawings of the celebrated Mascagni, from which the plates of his "Grand Anatomy" were taken, were finished in the year 1815, most of them as early as 1810; and that, among others who saw them in Italy in this state of preparation, Baron CUVIER may be particularly mentioned. He complains of the piracy committed upon this property by Professor ANTOMACHI, whose plates were identically the same with those of Mascagni; and the object of his discourse is to rectify this mistake, which had received some apparent sanction from an account of these plates published by the Institution in Paris.

---

### *New Association of Physicians.*

*To the Editors of the London Medical and Physical Journal.*

Gentlemen,—On reading your excellent Journal for the last month, it was with feelings of regret I observed a proposed plan for establishing a Society, called the "Society of Physicians of the United Kingdom," having for its particular object the advancement of the science of medicine.

Admitting the science of medicine might receive some further advancement from the combined efforts of its professors than individual exertion would be capable of effecting, yet would not that advantage be equally well, or indeed better, effected by the union of the larger body of the medical profession, the *general practitioners,* with the professors, than by the formation of a Society consisting only of comparatively few individuals, many of whom, although Fellows or Licentiates of the Royal College of Physicians, are scarcely known as men of practical experience, and hence can but seldom have opportunities of communicating useful and improved practical intelligence to their brother professors?

I am more particularly led to make the foregoing remarks, observing the 4th Resolution of this Society, viz. "the exclusion of all those who practise either *surgery, pharmacy,* or *midwifery.*" Now, Gentlemen, I much fear the consequence arising from the formation of such a Society: it will tend to place the general practitioner and physician at a greater distance from each other than they now are; consequently, materially affecting the professional recommendation of the latter; while the literary improvements of the former will be much injured by the absence of the eminent physician from the chairs of the Medical Societies, which have been established for years, and at which the general practitioner was accustomed, with pleasure, to meet his professional brethren, communicating personally any useful intelligence collected during his practice; and deriving the benefit arising from the opinions of those whom experience and long practice had raised to eminence; congratulating himself, at the same time, upon the handsome manner in which the different members of the profession voluntarily met each other, striving to further one great object, "the advancement of medical science."

I recollect, and with extreme pleasure I mention it, the distinguished Professor of Surgery at the Borough hospitals, told his class that he was indebted to their grandfathers and fathers, in a great measure, for his advancement in his profession, for affording him opportunities, by their patronage, of displaying that skill which, for a number of years, he has continued to practise with such unrivalled success. But, Gentlemen, we do not find him endeavouring to form a Surgical Society, the members of which must be regularly-educated surgeons only, to the prejudice of the practitioner of pharmacy and midwifery.

Now let me ask, to whom are the most celebrated physicians of this metropolis indebted for their advancement to their present eminence? whether or not from the information derived from association with, and patronage of, the general practitioners? And let me also ask, who is the best physician? he who spends seven years at a university in classical acquirements (which is the only one that can be admitted as a member of the Society of Physicians of the United Kingdom), or he who, fagging up the hill of fame as a general practitioner, becoming daily conversant with disease, at length reaches the summit of his profession,—not by merely possessing a diploma, but by the superior exercise of his professional talents when called upon.

I must now conclude, by observing that I cannot think the fourth object which this Society has in view, viz. the advancement of the *interests* and *dignity* of its professors, will ever be attained; for, whatever tends to separate the different members of a profession must certainly form a considerable barrier to the improvement in that profession, as well as to the pecuniary interests and dignity of its professors.

I trust, Gentlemen, to your liberality in inserting this letter in your valuable Journal, and beg to subscribe myself, Gentlemen, your most obedient servant, &c.

A GENERAL PRACTITIONER.

*London; August* 13*th*, 1824.

*** Had we even been enlisted among the members of the Association which our correspondent reprehends, we should not have laid claim to any great share of liberality in giving insertion to so temperate a protest.—EDITORS.

### METEOROLOGICAL JOURNAL,

*From July 20, to August 19, 1824.*

By Messrs. WILLIAM HARRIS and Co. 50, Holborn, London.

| | MOON. | Rain gauge | THERM. | | | BAROM. | | DE LUC'S HYG. | | WIND. | | ATMOSPHERIC VARIATIONS. | | |
|---|---|---|---|---|---|---|---|---|---|---|---|---|---|---|
| | | | 9 A.M. | MAX. | MIN. | 9 A.M. | 10P.M. | 9 A.M. | 10P.M. | 9 A.M. | 10P.M. | 9 A.M. | 2 P.M. | 10P.M. |
| July | | | | | | | | | | | | | | |
| 20 | | | 65 | 75 | 62 | 30.30 | 30.26 | 55 | 57 | NNE | N | Fine | Fine | Cloud. |
| 21 | | | 65 | 76 | 63 | 30.22 | 30.20 | 60 | 65 | NNE | SE | | | Fine |
| 22 | | | 67 | 73 | 64 | 30.21 | 30.16 | 61 | 65 | E | SE | | | |
| 23 | | | 67 | 72 | 59 | 30.15 | 30.02 | 60 | 53 | SW | SW | | | |
| 24 | | | 67 | 74 | 60 | 29.86 | 29.80 | 60 | 55 | W | W | | | |
| 25 | | | 72 | 70 | 58 | 29.80 | 29.83 | 53 | 55 | W | W | | | |
| 26 | ● | 5. | 67 | 70 | 56 | 29.80 | 29.80 | 60 | 70 | NE | NE | | Cloud. | Rain |
| 27 | | | 63 | 70 | 62 | 29.94 | 30.14 | 70 | 55 | NNE | NE | Cloud. | Fair | Fair |
| 28 | | | 65 | 70 | 60 | 30.23 | 30.20 | 53 | 58 | SW | SW | Fine | Fine | Fine |
| 29 | | | 67 | 70 | 56 | 30.07 | 29.87 | 59 | 65 | ESE | E | | | |
| 30 | | | 57 | 69 | 53 | 29.64 | 29.60 | 65 | 67 | E | E | Fair | Fair | Fair |
| 31 | | | 57 | 67 | 53 | 29.67 | 29.60 | 70 | 79 | E | E | Fine | Cloud. | Rain |
| Aug. | | | | | | | | | | | | | | |
| 1 | ☽ | .32 | 58 | 63 | 50 | 29.60 | 29.95 | 85 | 75 | NE | N | Rain | Rain | Fine |
| 2 | | | 57 | 69 | 53 | 30.03 | 30.00 | 70 | 77 | NW | SW | Fine | Fine | |
| 3 | | | 65 | 69 | 57 | 29.95 | 29.91 | 75 | 73 | SSW | S | Cloud. | | Cloud. |
| 4 | | | 66 | 68 | 56 | 29.84 | 29.75 | 70 | 75 | S | W | Fair | | Rain |
| 5 | | | 58 | 68 | 57 | 29.70 | 29.68 | 71 | 74 | WSW | SW | Fine | Fine | Fine |
| 6 | | .33 | 60 | 65 | 53 | 29.61 | 29.68 | 70 | 77 | WSW | W | Cloud. | Rain | Rain |
| 7 | | | 57 | 67 | 54 | 29.83 | 29.90 | 75 | 77 | NNW | N | Overc. | | |
| 8 | | | 55 | 65 | 54 | 29.85 | 29.73 | 75 | 87 | W | SW | Rain | | |
| 9 | ○ | | 57 | 70 | 50 | 29.70 | 29.78 | 85 | 73 | W | W | Fine | Fine | Fine |
| 10 | | | 65 | 70 | 54 | 29.82 | 29.78 | 62 | 77 | W | S | | | |
| 11 | | | 65 | 72 | 59 | 29.65 | 29.73 | 70 | 70 | W | WSW | Cloud. | | |
| 12 | | | 66 | 70 | 55 | 29.76 | 29.77 | 67 | 73 | WSW | W | Fair | | |
| 13 | | | 69 | 68 | 53 | 29.83 | 29.84 | 70 | 72 | W | N | Fine | Sho'ry | |
| 14 | | | 64 | 68 | 55 | 29.96 | 29.95 | 68 | 70 | W | S W | | Fine | Fair |
| 15 | | 145 | 66 | 67 | 53 | 29.74 | 29.70 | 80 | 78 | SW | W | Rain | Sho'ry | |
| 16 | | | 63 | 67 | 53 | 29.70 | 29.80 | 70 | 70 | W | W | Fine | Fine | Fair |
| 17 | ☾ | | 64 | 68 | 55 | 29.80 | 29.63 | 70 | 73 | WSW | WSW | | Fair | |
| 18 | | | 65 | 67 | 51 | 29.64 | 29.57 | 70 | 70 | SW | W | | Rain | Cloud. |
| 19 | | .16 | 64 | 65 | 55 | 29.74 | 29.80 | 65 | 78 | W | SW | | Fair | Rain |

The quantity of rain fallen in the month of July, was 1 inch and 35.100ths.

